# Global challenges of graduate level Ayurvedic education: A survey

**DOI:** 10.4103/0974-7788.59945

**Published:** 2010

**Authors:** Kishor Patwardhan, Sangeeta Gehlot, Girish Singh, H.C.S. Rathore

**Affiliations:** *Department of Kriya Sharir,Banaras Hindu University, Varanasi, India*; 1 *Department of Community Medicine,Banaras Hindu University, Varanasi, India*; 2 *Faculty of Education, Banaras Hindu University, Varanasi, India*

**Keywords:** Ayurveda education, global challenges, India, mailed survey

## Abstract

In the present day scenario, Ayurveda is globally being perceived in several contradictory ways. Poor quality of Ayurveda graduates produced as a result of poorly structured and poorly regulated education system is at least one of the important factors responsible for this scenario. The present study was carried out to evaluate the ‘Global challenges of graduate level Ayurvedic education’ and is based on the responses of Ayurvedic students and Ayurvedic teachers from various educational institutions of India to a methodically validated questionnaire. As the study indicates, the poor standard of Ayurvedic education in India is definitely a cause of concern. The curriculum of Bachelor of Ayurvedic Medicine and Surgery (BAMS) course of studies is required to be reviewed and restructured. The syllabi are required to be updated with certain relevant topics like laws governing the intellectual property rights, basic procedures of standardization of medicinal products, fundamental methods of evaluating the toxicity of the medicinal products, essentials of healthcare management and the basics of cultivation and marketing of medicinal plants. Furthermore, the study suggests that the Ayurvedic academicians are required to be trained in standard methods of research and documentation skills, and the educational institutions are required to be encouraged to contribute their share in building up the evidence base for Ayurveda in the form of quality education and research.

## INTRODUCTION

Perceptions about Ayurveda in India and overseas have undergone a phenomenal change during the last 20 to 25 years. A large population from all over the world is attracted towards this ancient system of healthcare because of the terms associated with it like ‘Holistic Medicine’, ‘Herbal’, ‘Free from side effects’, ‘Mind, Body and Spiritual approach’ etc. Centers offering ‘*Panchakarma* therapy’, ‘Ayurvedic Lifestyle Management’ and ‘Ayurvedic Massage’ are being increasingly established. The use of Ayurvedic medicines has become accepted in other countries as well. For example, according to the 2007 National Health Interview Survey, more than 200 000 US adults had used Ayurvedic medicine in 2006 alone.[1] Marketing strategies of major pharmaceutical firms have changed and ‘Ayurvedic wings’ of drug manufacturing have begun. In 2007, there were more than 8400 licensed Ayurvedic pharmacies in India and the approximate turnover of this industry was Rs. 4000 crore, which accounted for nearly a third of the total pharmaceutics business of the country.[2] Many medical schools and other institutes all over the world have started offering some degree or diploma in Ayurveda. Several publication houses of international repute have started publishing literature related to Ayurveda. Even the people with no formal Ayurvedic education have started showing interest in authoring books and research papers on Ayurveda.[3] Ayurveda is being seen as a rich resource for new drug development by modern day pharmacologists.[4]

On the contrary, questions on safety and efficacy of Ayurvedic products are also being raised.[5,6] In 2004 December, Journal of American Medical Association (JAMA) published a research paper, which concluded that one of the five Ayurvedic Herbal Medicine Products (HMPs) produced in South Asia and available in Boston South Asian grocery stores contained potentially harmful levels of lead, mercury and/or arsenic. The paper also suggested that the users of Ayurvedic medicine may be at risk for heavy-metal toxicity, and testing of Ayurvedic HMPs for toxic heavy metals should be made mandatory.[7] This concern has led some countries like Canada to curb the import of Ayurvedic preparations from India. In 2005, the testing by Canadian Government revealed alarmingly high levels of heavy metals in the exported Ayurvedic medicinal preparations. The analysis highlighted the ‘higher than acceptable concentrations’ of heavy metals such as leadmercury and arsenic.[8] A similar paper appeared in JAMA in 2008 too, raising an alarm against the use of Ayurvedic products because of their possible heavy-metal contamination.[9] National Policy on Indian Systems of Medicine and Homeopathy, 2002 has also admitted that the safety, efficacy, quality of drugs and their rational use have not been assured in India. This document states that there is no assurance whatsoever that formularies and pharmacopeial standards are being followed by the Indian Systems of Medicine drug manufacturers.[10]

ThusAyurveda is globally being perceived in several contradictory ways. Poor quality of Ayurveda graduates produced as a result of a poorly structured and poorly regulated education system is at least one of the important factors responsible for this scenario. The number of Ayurveda colleges has increased phenomenally to 242, out of which, about 150 colleges have been established after 1980. Though the Central Council of Indian Medicine (CCIM) has implemented various educational regulations to ensure minimum standards of education, there has been a mushroom growth of sub-standard colleges causing erosion to the standards of education. Liberal permission by the State Governments, loopholes in the existing Acts and weakness in the implementation of standards of education have been held responsible for this state of affairs.[11]

Considering the above facts, the present study was planned to evaluate the global challenges of graduate level Ayurvedic education. The study is based on the perceptions of Ayurvedic students and Ayurvedic teachers from various educational institutions spread all over India.

## STUDY DESIGN

### Method

Mailed survey was the method that was used to carry out the present study.

### Population

The population for the present study was defined in terms of the students studying and teachers teaching in Ayurvedic colleges of India during the period of September 2005 to October 2008.

### Inclusion criteria

All interns/house surgeons registered under Bachelor of Medicine and Surgery (BAMS) course, who have successfully passed their third professional BAMS examination in the Ayurvedic colleges recognized by CCIM were included in the study. Students who have not yet passed their third professional examinations were excluded from the study to avoid possible immature and biased perceptions.

All Post-graduate students registered for MD(Ay) or MS(Ay) courses in the Ayurvedic colleges recognized by CCIM were also included in the study.

All teachers working in Ayurvedic colleges / universities recognized by CCIMwho possess at least a BAMS or an equivalent degreewere included in the study.

### Sample

The sample frame that became available constituted a list of 242 Ayurvedic colleges spread all over India. As the students and teachers in these colleges formed the primary units of sampling, it was essential to get a list of all these students and teachers for the purpose of randomization. But, as no such database is available in Indiathe list of 242 Ayurvedic colleges was accepted as the sample frame for this study. With the availability of the said sample frame, the Random Cluster Sampling technique was considered to be the most appropriate one. Henceit was decided to include at least 10% of the total institutions present in a specific geographical zone of India (North, East, South and West). At the same timean effort was made to include as many states as possible. The colleges from each geographical zone were selected randomly and all the teachers and students from these collegesas per the operational definition stated earlierwere taken as the clusters to constitute the sample. Thus, a total of 32 colleges were included in the study.

### Preparation of the Questionnaire

A preliminary list of items was prepared on the basis of interactions we had with the students and teachers of several educational institutions. Diverse sources of literature like reports of various committees, journals, news reports, national health policy documents and other articles were referred to for collecting the items. The questionnaire comprised of three sections dealing with three problem areas, viz.‘Job Opportunities’, ‘Global challenges’ and ‘Entrepreneurship opportunities’. The respondents were given the option of recording their responses in the form of ‘Strongly Agree’, ‘Agree’‘Undecided’‘Disagree’ and ‘Strongly Disagree’ by recording a check mark (√) in the respective column provided for the purpose. Apart from these statements, the respondents were also asked to furnish some demographic details such as their agegenderinstitutional affiliation and the present status (student or teacher).

### Validation of the Questionnaire for its Reliability and Consistency

The preliminary questionnaire was distributed to 150 respondents who randomly fulfilled the inclusion criteria in a single institution. The validation process of the questionnaire was carried out on the basis of the first 100 completed questionnaires. The data entry was done using the software ‘Statistical Package for Social Sciences’, Version 11.5 (SPSS Inc. Chicago, IL, USA). Demographic data were fed in the ‘String’ format and the responses to the questionnaire were entered in the ‘Numerical’ format. For the purpose of conversion of responses into the numerical format, the following scoring system was used: Strongly Agree=5; Agree= 4; Undecided=3; Disagree=2 and Strongly Disagree=1.

For the purpose of validationeach section of the questionnaire was considered as an independent scale and these scales were tested for their reliability using ‘Cronbach's Coefficient Alpha’.[12] While validating the scalesvalue of alpha greater than 0.7 and item-total correlation greater than 0.2 were considered to be acceptable.[13] Wherever the value of ‘Alpha (when item deleted)’ was greater than Cronbach's coefficient alphathe corresponding item was deleted and the whole process of validation was repeated and thereafter, the scales were finalized.

The final questionnaire contained three sections (technically, three independent scales) comprising of a total of 24 items. Apart from these items, the questionnaire also contained a copy of confidentiality agreement stating the purpose of the study and assuring strict confidentiality of the respondents.

### Collection of Data

After validation and testing the questionnaire for its reliabilitythe questionnaire was printed on the A4-sized paper and was mailed to 32 Ayurvedic colleges. Varying numbers of questionnaires were mailed to different institutions considering the total admission capacity, the total number of teachers presentpresence or absence of post-graduate courses etc. The heads of the institutes were requested through a formal letter to distribute the questionnaire among internspost-graduate students and teachers. A self-addressed stamped envelope was also sent in order to facilitate the return of the completed questionnaires. A period varying from 1 to 2 days was given to the respondents to return the completed questionnaires. The completed questionnaires were then collected and the data entry was carried out as explained earlier.

### Response Rate

The response rate for the student group was 59.6% and for the teacher group it was 54%. A total of 1022 participants from 18 states responded to the questionnaire. This number included 644 students and 378 teachers.

### Tabulation and Statistical Tests

The participants were grouped under two categories: ‘Students’ and ‘Teachers’. Tables of frequency and percentage were framed on the basis of responses to individual items for each group. Independent samples - *t* test was applied to compare the mean scores of the two groups.

## RESULTS

Tables 1–3 summarize the results of the present study. Tables show the items covered in the final questionnaire along with the mean scores for both the groups separately. Also, the results of independent samples - *t* test are shown in the form of *τ* values and *p* values. As per the scoring pattern followed in the study, the mean scores greater than 3 indicate a tendency towards agreementwhereas the mean scores lesser than 3 indicate a tendency towards disagreement. Furthermorethe mean scores greater than 4 indicate a strong tendency towards agreement. As the tables showmean scores for both the groups are greater than 3 for all the 24 items indicating a general tendency towards agreement.

**Table 1:** Responses of the participants to the items related to the ‘Job Opportunities after BAMS course’. The mean scores for both the groups are given along with the results of Independent samples-t test.

	Items	Group	Mean±SD	t	p
Q1.1	Legally, in most of the states, a BAMS degree holder cannot practice Allopathy and therefore hospitals generally prefer MBBS graduates as medical offi cers instead of BAMS graduates.	Students	439 ± 0.945	6.732	0.000
		Teachers	3.94 ± 1.142		
Q1.2	Ayurvedic hospitals are less in number in comparison to Allopathic ones and therefore job opportunities are limited.	Students	4.41 ± 0.876	2.415	0.016
		Teachers	4.27 ± 0.881		
Q1.3	In Ayurvedic educational institutionsonly Post-Graduate doctors are employed and not BAMS degree holders.	Students	4.15 ± 1.064	4.717	0.000
		Teachers	3.81 ± 1.165		
Q1.4	Most of the research institutions prefer Post-Graduate doctors and thereforejob opportunities in research institutions are limited.	Students	4.34 ± 0.830	2.866	0.004
		Teachers	4.19 ± 0.831		
Q1.5	Even in government sectorBAMS graduates are not treated at par with MBBS graduates and thereforejob opportunities are limited in certain areas e.g. Railways.	Students	4.69 ± 0.610	3.808	0.000
		Teachers	4.53 ± 0.718		
Q1.6	Ayurvedic pharmaceutical firms prefer Post-Graduate candidates to BAMS degree holders as experts.	Students	4.28 ± 0.803	3.476	0.001
		Teachers	4.09 ± 0.898		
Q1.7	There is lot of competition for jobs among BAMS degree holders as a result of mushrooming of Ayurvedic colleges.	Students	4.21 ± 0.955	1.140	0.255
		Teachers	4.14 ± 1.010		

**Table 2:** Responses of the participants to the items related to the ‘Global Issues concerned to Ayurveda’. The mean scores for both the groups are given along with the results of Independent samples-t test.

	Item	Group	Mean ±SD	t	p
Q2.1	Serious questions are being raised on the safety profile of Ayurvedic preparations in some countries posing a threat to the Ayurvedic system of Medicine.	Students	4.27 ± 0.867	4.496	0.000
		Teachers	4.00 ± 1.013		
Q2.2	Standardization of Ayurvedic preparations is still a problem that needs to be addressed.	Students	4.47 ± 0.706	3.338	0.001
		Teachers	4.31 ± 0.738		
Q2.3	In many countrieslegallypracticing Ayurveda is not allowed and thereforethere are no opportunities for BAMS graduates in such countries.	Students	4.40 ± 0.833	2.380	0.018
		Teachers	4.27 ± 0.792		
Q2.4	Possible entry of foreign universities in India may pose a threat to the existing educational institutions.	Students	3.77 ± 1.158	3.460	0.001
		Teachers	3.51 ± 1.202		
Q2.5	Ayurvedic academicians do not figure anywhere in authoring the scientific and evidence-based papers in reputed international journals.	Students	3.95 ± 1.081	1.490	0.136
		Teachers	3.84 ± 1.081		
Q2.6	Ayurvedic academicians do not voluntarily participate in International platforms to present their research data.	Students	3.79 ± 1.131	0.756	0.450
		Teachers	3.73 ± 1.138		
Q2.7	Ayurvedic academicians do not follow international standards while planning the protocols of research projects and while writing research reports.	Students	3.86 ± 1.045	-0.095	0.924
		Teachers	3.87 ± 1.020		
Q2.8	Ayurvedic scholars generally do not have knowledge regarding'Intellectual Property Rights' and patenting procedures.	Students	3.97 ± 0.943	0.824	0.410
		Teachers	3.92 ± 0.975		
Q2.9	Authentic websites providing up-to-date knowledge in Ayurveda are not hosted by Ayurvedic institutions.	Students	4.20 ± 0.918	0.073	0.942
		Teachers	4.19 ± 0.769		
Q2.10	No standard international indexed and peer-reviewed journals are published by Ayurvedic institutions making it difficult for Ayurvedic researches have global attention.	Students	4.19 ± 0.885	0.628	0.530
		Teachers	4.15 ± 0.853		
Q2.11	Pharmacodynamic/ pharmacokinetic properties/efficacy/safety profiles and chemical compositions of Ayurvedic formulations are yet to be established making it difficult for experts in conventional medicine to accept Ayurveda.	Students	4.25 ± 0.893	1.896	0.058
		Teachers	4.15 ± 0.809		

**Table 3:** Responses of the participants to the items related to the ‘Entrepreneurship /Business opportunities’ after the BAMS course. The mean scores for both the groups are given along with the results of Independent samples-t test.

	Item	Group	Mean± SD	t	p
Q3.1	Students are not trained in management skills required to launch a new Ayurvedic hospital/Panchakarma center/Ayurvedic Pharmacy during BAMS course.	Students	4.18 ± 1.051	2.170	0.030
		Teachers	4.03 ± 0.981		
Q3.2	Students are not exposed to the basics of economical aspects related to healthcare sector during BAMS course.	Students	4.18 ± 0.890	2.078	0.038
		Teachers	4.06 ± 0.856		
Q3.3	Most of the BAMS graduates prefer either studying PG course or they go for private practice and thereforeinspiring examples of industrially successful BAMS graduates are very few.	Students	4.38 ± 0.750	5.693	0.000
		Teachers	4.09 ± 0.840		
Q3.4	Students are not introduced to the skills related to the management of Health tourism and emerging opportunities in this field during BAMS course.	Students	4.35 ± 0.782	2.452	0.014
		Teachers	4.23 ± 0.776		
Q3.5	Students are not exposed to the agricultural and marketing aspects of medicinal plants making it difficult to go for cultivation/marketing of medicinal plants.	Students	4.39 ± 0.690	3.430	0.001
		Teachers	4.22 ± 0.800		
Q3.6	Students are not exposed to the manufacturing techniques related to cosmetic products and such other popular dosage forms during BAMS course making them unfit for modern pharmaceutical industry.	Students	4.34 ± 0.867	3.102	0.002
		Teachers	4.16 ± 0.864		

## DISCUSSION

### Job opportunities

The observations related to this section [Table 1] of the questionnaire indicate that there is a real problem related to the job opportunities for BAMS graduates. Except for Q1.2 and Q1.7, the tendency for agreement towards all the items is significantly stronger among students than the teachers as indicated by *p* values. This means that students perceive this problem to be a more serious one in comparison to teachers. This indicates that there is a considerable level of career-related anxiety among students. This anxiety is noticeably less among teachers because they are already into a job.

The Government is required to look into the matter related to the creation of job opportunities for BAMS graduates in certain departments like Railways and Defence. In teaching institutions too, some posts like tutors and medical officers may be created for BAMS graduates. Ayurveda may be included as an optional subject in the entrance examinations leading to Indian Administrative Services (IAS) just like modern medicine. If the quality of education is improved, some job opportunities may open up in research institutes and in other places in the healthcare industry as well.

### Global Issues

As the Table 2 shows, both the students and the teachers show a strong tendency towards agreement that the issues related to safety profile and standardization of Ayurvedic products are serious ones. A majority of students and teachers also tend to agree that in many countries, practicing Ayurveda is not legally allowed and thereforethere are no opportunities for BAMS graduates in such countries. Also, there is a general tendency towards agreement that Ayurvedic academicians do not figure anywhere in authoring the scientific and evidence-based papers in reputed international journals and they do not voluntarily participate in international platforms to present their research data. The study also suggests that Ayurvedic academicians do not follow international standards while planning the protocols of research projects and while writing research reports. Ayurvedic scholars generally do not have knowledge regarding ‘Intellectual Property Rights’ and patenting procedureswhich is another issue that this study is indicative of.

The reasons for such perceptions need to be explored. The current curriculum of BAMS does not include the relevant and essential topics like laws governing the intellectual property rights, patenting proceduresbasic methods of standardization of medicinal productsfundamental principles of evaluating the toxicity of the medicinal products and basics of pharmacovigilance. Experts in phytochemistry, pharmacognosy, pharmacology, biotechnology and other relevant fields may be appointed in teaching institutions as teachers to teach these topics. Basics of research methodology are also not introduced in the present BAMS course of study. Some essential information related to these topics may be introduced in the curriculum making BAMS graduates conversant in these topics. Training programs and workshops may be required to be introduced for Ayurvedic academicians where, training may be given in planning the research protocolspreparing the research projects and in other various areas of research methodology.

A very significant proportion of the students and the teachers in the present study tend to agree that authentic websites providing recent advances in Ayurveda are not hosted by Ayurvedic institutions. In this regard, the education institutions are required to be encouraged to host authentic websites giving information related to various aspects of Ayurveda. Classical textbooks of Ayurveda may also be made available online at these websites along with the information related to recent advances. The Government also may take initiative in this regard and launch authentic websites.

A significant number of participants in the study tend to agree that no standard international indexed and peer-reviewed journals are published by Ayurvedic institutions making it difficult for Ayurvedic researches have global attention. However, recently, National Institute of Ayurveda (NIA), Jaipur and Institute of Post-Graduate Teaching and Research in Ayurveda (IPGTandRA), Jamnagar have launched their own peer-reviewed journalswhich is a positive sign. But their reach is limited by the fact that online versions of these journals are not available. The department of AYUSH has recently taken initiative in this regard by launching the International Journal of Ayurveda Research (IJAR), which is an online journal.

A majority of students and teachers in the present study tend to agree that Pharmacodynamic / pharmacokinetic properties/efficacy/safety profiles and chemical compositions of Ayurvedic formulations are yet to be established making it difficult for experts in conventional medicine to accept Ayurveda. As a solutionthe basic topics related to these issues may be incorporated into the curricula of BAMS and post-graduate courses of Ayurveda making the graduates and post- graduates of Ayurveda efficient in carrying out such studies.

### Entrepreneurship/Business Opportunities

So far, unlike conventional biomedicine or Allopathy, pharmaceutics has been a part of Ayurvedic education in India in the form of ‘Rasashastra’. There is a need of training to be imparted in the basic knowledge related to pharmaceutical industry during graduate level Ayurvedic education. Furthermore, Ayurvedic pharmaceutics is based on a large number of medicinal plants and the basic knowledge related to the cultivation and marketing of these plants is required to be passed on during Ayurvedic education. This will make an Ayurvedic graduate have a lot of opportunities other than private practice. But, as the Table 3 suggests, such entrepreneurship opportunities are scarce for BAMS graduates because of poor training during their Ayurvedic education. As a solution, some basic management skills that are essentially required to launch a new Ayurvedic hospital / Panchakarma center / Ayurvedic Pharmacy etc., may be included in the curriculum. Also, basic cultivation and marketing aspects of medicinal plants are needed to be introduced at BAMS level. Some manufacturing techniques related to cosmetic products and such other popular dosage forms may also be introduced. Introductory lessons in the management of health tourism may also be incorporated during the graduate level Ayurvedic education.

## CONCLUSION

As the present study indicates, the poor standard of graduate level Ayurvedic education in India is definitely a cause of concern. The curriculum of BAMS course of studies is required to be reviewed and restructured. The syllabi are required to be updated with certain relevant topics like laws governing the intellectual property rightsbasic procedures of standardization of medicinal productsfundamental methods of evaluating the toxicity of the medicinal productsessentials of healthcare management and the basics of cultivation and marketing of medicinal plants. Ayurvedic academicians are required to be trained in standard methods of research and documentation skillsand the educational institutions are required to be encouraged to contribute their share in building up the evidence base for Ayurveda in the form of quality education and research.
